# Preliminary screening of microplastic contamination in different marine fish species of Taif market, Saudi Arabia

**DOI:** 10.1515/biol-2022-0034

**Published:** 2022-04-06

**Authors:** Yassir Khattab, Amaal Mohammadein, Jamila S. Al Malki, Nahed Ahmed Hussien, Ehab M. Tantawy

**Affiliations:** Department of Biology, College of Science, Taif University, P.O. Box 11099, Taif 21944, Saudi Arabia; Research and Development Sector, EGYVAC, VACSERA, Giza 12311, Egypt

**Keywords:** fish, FTIR, KSA, microplastics, Nile red, Taif

## Abstract

Microplastics (MPs), as a physical anthropogenic contaminant, represent a serious, human health concern due to their toxicity and ability to act as vectors for other pollutants and pathogens. This study aimed to screen for MP contamination in marine fish in Taif market, Saudi Arabia. A total of 22 fish species were used according to their different marine habitats and feedings. We have focused on extracting MPs from gills and muscles using KOH digestion. Nile red dye was used for the MP identification under fluorescence microscopy followed by the Fourier-transform infrared spectroscopy analysis. This study has reported MP contamination in gills and muscles of all the studied fish, in which poly(vinyl butyral) (PVB) was present in epipelagic species, poly(vinylidene fluoride) (PVDF) and poly(2,4,6,-tribromostyrene) (PtBS) were present in pelagic species, and PtBS and chlorosulfonated polyethylene were present in demersal/benthopelagic species. Moreover, benthic fish samples contain PtBS particles; reef-associated species have three different MP particles/fiber PtBS, PVDF, and poly(vinyl formal) and the rest of the studied species samples contain PtBS. The results highlight that the MP pollution increased to reach different species from the pelagic species to the benthic ones. PtBS as a type of polystyrene was the most dominant MP found in most species.

## 1 Introduction

A plastic litter with various sizes and macro-, meso, micro-, and nanoscales is widely spread worldwide and has become heavily accumulated in our environment [1,2,3]. As a result, plastics have become the most dominant part of marine litter. In 2014, it was recorded that at least 5.25 trillion of plastic wastes (about 268,000 tons) have been discarded into the oceans [4]. By 2021, Meijer et al. [5] have estimated that about 0.80–2.7 million metric tons of macroplastics (their size is larger than 5 mm) enter the global oceans annually. Moreover, there is a dramatic increase in plastic litter entering both terrestrial and marine environments during the current COVID-19 pandemic [6]. Plastics were broken down into smaller microplastic (MP) fragments by ultraviolet radiation, mechanical forces, or oxidation [7]. MPs are found in different marine zones, from the surface to the water column and at the bottom [8,9].

It is not easy to specify the source of MP contamination in the marine environment, because everything is now made of plastics. However, it was recorded that coastal cities, coastal landfills, coastal dumping sites, shipping activities, ship coatings, binders of marine paints, and ports are the most critical sources of plastic contamination in the marine environment [10,11,12]. In addition, because of MPs’ small size, they could be accidentally ingested by filter feeders and a broad range of organisms that in turn could threaten ecosystems, including human health [13,14,15].

MPs have been detected in a wide range of marine organisms from various trophic levels [6]. Different research studies have reported MPs in the alimentary canal and gills of various marine and freshwater fish species, shellfish, and crustaceans. Without removing their gastrointestinal tract, shellfish and small fish are consumed by humans that might give the most explicit introduction of MPs via meal. In contrast, this is not usual in big fish preparation for human consumption, in which gastrointestinal tract, gills, scales, skin, and fins are removed before their consumption that could decrease their risk on human health [16], but the risk is the entrance of MPs to other tissues, especially the edible muscles of fish. In our peer knowledge, this point has not been well studied until now.

In 2017, Marti et al. [17] have reported that the Saudi Arabian coast of the Red Sea has a much lower load of floating MPs than expected based on its nature as a semi-enclosed sea with an inverted estuarine circulation. They stated that the main sources of MP contamination are synthetic fibers from the rope, packaging materials, and washing of synthetic clothing that are derived from land inputs with sewage and wastewater, or atmospheric deposition. Recently, Baalkhuyur et al. [18] have assessed the presence of MPs in the gastrointestinal tract of 26 commercial and noncommercial fish species from 4 different habitats sampled along the Saudi Arabian coast of the Red Sea. They have reported by using the Fourier-transform infrared spectroscopy (FTIR) analysis that polypropylene and polyethylene are the most popular extracted polymers found in their study samples. In addition, *Epinephelus* sp. (benthic species) that was sampled in the Jazan region registered the highest number of ingested MPs. They have concluded that the higher abundance of MPs in fish samples may be related to their marine habitat and the abundance of MP debris near the seabed.

The present study aimed to screen for MP contamination in gill and muscle samples of different marine fish species in markets of the Taif governorate of Saudi Arabia. Several species were selected to have a wide range according to their marine habitat and feeding. Nile red dye was used to detect MPs with three different sets of filters for fluorescence microscope examination and then analyzed by FTIR to report their type.

## 2 Materials and methods

### 2.1 Fish collection

**Figure 1:**
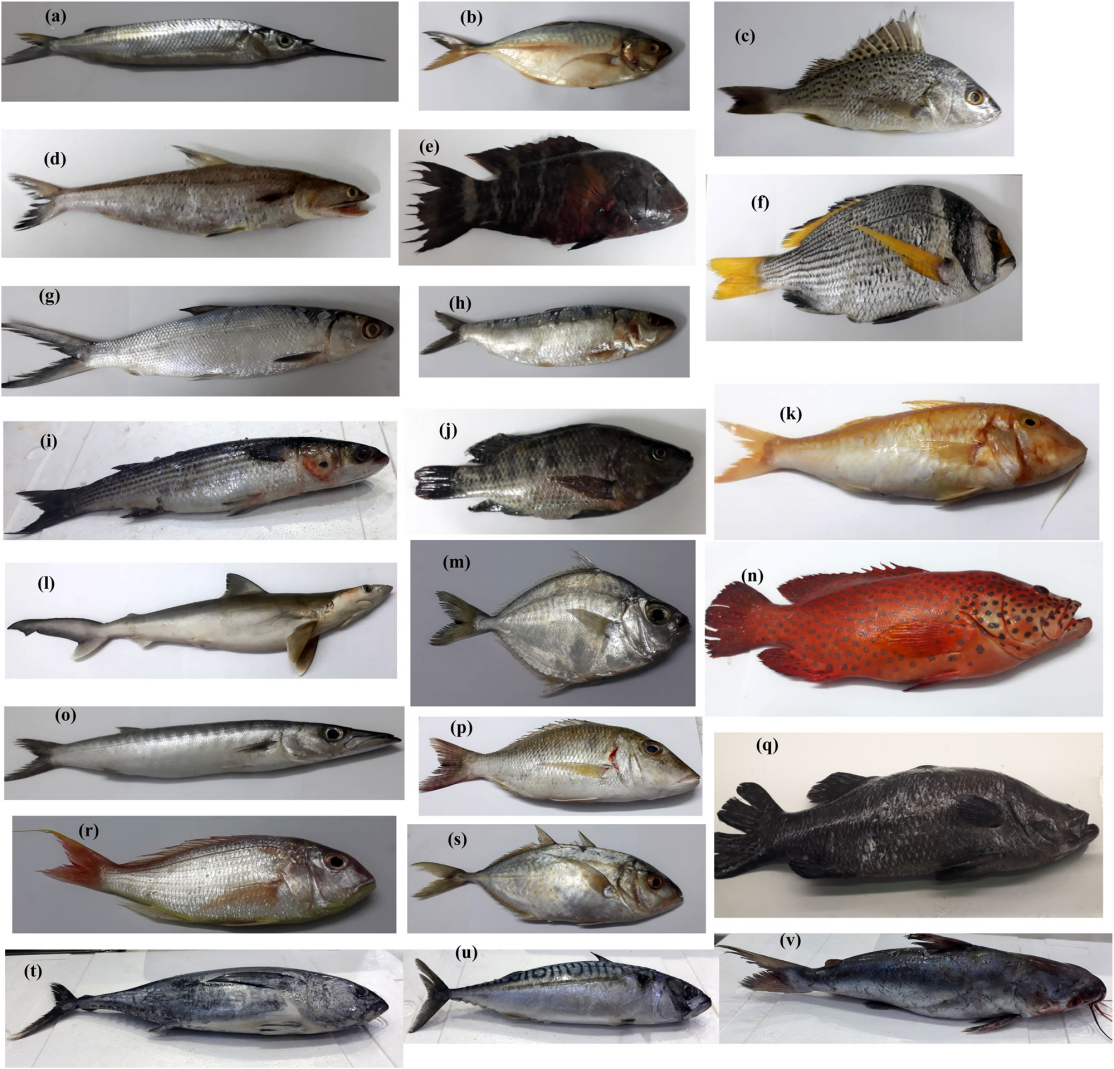
Different fish species were collected from the Taif market for the present study, in which (a) *Hemiramphus far*, (b) *Trachurus indicus*, (c) *Pomadasys argenteus*, (d) *Saurida undosquamis*, (e) *Calotomus viridescens*, (f) *Acanthopagrus catenula*, (g) *Chanos chanos*, (h) *Sardina pilchardus*, (i) *Mugil cephalus*, (j) *Oreochromis spilurus*, (k) *Mullus barbatus*, (l) *Squalus acanthias*, (m) *Pampus argenteus*, (n) *Epinephelus morio*, (o) *Sphyraena barracuda*, (p) *Lethrinus nebulosus*, (q) *Centropristis striata*, (r) *Pagrus major*, (s) *Caranx caninus*, (t) *Thunnus orientalis*, (u) *Scomber scombrus*, and (v) *Netuma thalassina.*

A total of 22 different fish species samples (*n* = 1), *Hemiramphus far*, *Trachurus indicus*, *Pomadasys argenteus*, *Saurida undosquamis*, *Calotomus viridescens*, *Acanthopagrus catenula*, *Chanos chanos*, *Sardina pilchardus*, *Mugil cephalus*, *Oreochromis spilurus*, *Mullus barbatus*, *Squalus acanthias*, *Pampus argenteus*, *Epinephelus morio*, *Sphyraena barracuda*, *Lethrinus nebulosus*, *Centropristis striata*, *Pagrus major*, *Caranx caninus*, *Thunnus orientalis*, *Scomber scombrus*, and *Netuma thalassina*, were collected from Taif market, Saudi Arabia (Figure 1). Table 1 shows the marine and feeding habitats of the studied fish species according to Froese and Pauly [19]. Fish were kept on ice before dissection. Each fish species was dissected separately; muscles and gills were exited. For each fish, about 1 g of gills and muscles were used independently for MP extraction.

**Table 1:** Fish different species used in the present study showing their marine and feeding habitats [19]

Species name	Common name	Marine habitat	Feeding habitat
*Hemiramphus far*	Halfbeak	Reef-associated	Algae, zooplankton, small fishes
*Trachurus indicus*	Arabian scad	Benthopelagic	Fish fry and small crustaceans
*Pomadasys argenteus*	Silver grunt	Coastal inshore waters	Small invertebrates
*Saurida undosquamis*	Brushtooth lizardfish	Reef-associated	Fishes, crustaceans, and other invertebrates
*Calotomus viridescens*	Viridescent/dotted parrotfish	Reef-associated	Marine angiosperms and epiphytic algae
*Acanthopagrus catenula*	Bridled seabream	Reef-associated	Bivalves and shrimps
*Chanos chanos*	Milkfish	Demersal (benthopelagic)	Soft algae, small benthic invertebrates, and pelagic fish eggs and larvae
*Sardina pilchardus*	European pilchard	Epipelagic	Planktonic crustaceans and larger organisms
*Mugil cephalus*	Flathead gray mullet	Pelagic	Detritus, microalgae, and benthic organisms
*Oreochromis spilurus*	Sabaki tilapia	Shallow water	Insects, plants, and diatoms
*Mullus barbatus*	Red mullet	Benthic	Small benthic crustaceans, worms, and mollusks
*Squalus acanthias*	Spiny dogfish	Demersal (benthopelagic)	Diversity of prey, ranging from comb jellyfish, squid, mackerel, benthic fishes, shrimps, crabs, and sea cucumbers
*Pampus argenteus*	Silver pomfret	Demersal (benthopelagic)	Ctenophores, salps, medusae, and other zooplankton
*Epinephelus morio*	Red grouper	Reef-associated	A wide variety of fishes and invertebrates
*Sphyraena barracuda*	Great barracuda	Pelagic	Fishes, cephalopods and sometimes on shrimps
*Lethrinus nebulosus*	Spangled emperor	Reef-associated	Echinoderms, mollusks, crustaceans, polychaetes, and fish
*Centropristis striata*	Black sea bass	Demersal (benthopelagic)	Crabs, shrimps, barnacles, worms, tunicates, small fish, and bivalves
*Pagrus major*	Red seabream	Demersal (benthopelagic)	Benthic invertebrates, including echinoderms, worms, mollusks, and crustaceans; also, on fishes
*Caranx caninus*	Pacific crevalle jack	Benthic	Mainly fishes, but also takes shrimps and other invertebrates
*Thunnus orientalis*	Pacific bluefin tuna	Pelagic	A wide variety of small schooling fishes and squids, also on crabs
*Scomber scombrus*	Atlantic mackerel	Pelagic	Zooplankton and small fish
*Netuma thalassina*	Giant catfish	Demersal (benthopelagic)	Crabs, prawns, mantis shrimps (*Squilla* species) but also on fishes and mollusks

### 2.2 Sample preparation and MP extraction

**Figure 2:**
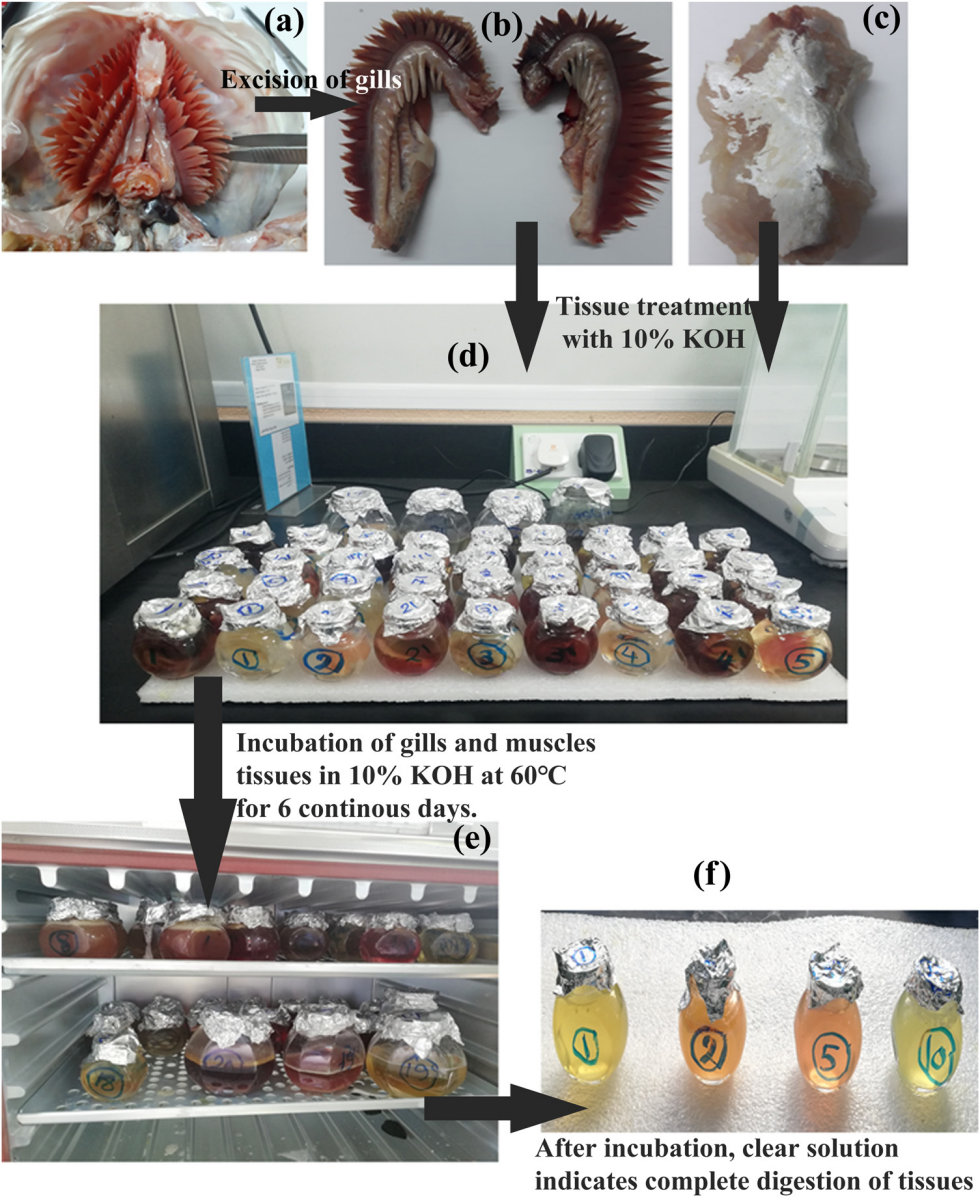
Steps of MP extraction from gills (a and b) and muscle (c), in which, image (d) refers to tissues’ treatment with 10% KOH and incubation at 60°C (e) and then left for 6 days until complete digestion (f).

The gills and muscles of each fish were subjected to caustic (KOH) digestion separately. According to Rochman et al. [20], with few modifications, each sample was incubated in aqueous KOH (10%) for 6 days at 60°C with agitation until complete digestion. The colorless KOH turned yellow, red, or pale brown by the end of digestion without any remaining organic residues. Next, each sample was filtered through 8 µm Whatman^®^ Grade 2 cellulose filters, and then filters were rinsed in deionized water into glass Petri dishes. Finally, filters were kept at 60°C for complete dryness, and then dry filters were collected per sample for further evaluation (Figure 2).

### 2.3 Detection of MPs

The dried filters of each sample were loaded on glass slides and stained with a few drops of 1 mg/mL Nile red dye in acetone. Slides were kept in the dark box for 20 min before fluorescence microscope examination, according to Sturm et al. [21] with few modifications. The fluorescence photomicrographs were taken with Zeiss Imager Z.1 (Carl Zeiss Microscopy GmbH, Jena, Germany) in epifluorescence mode (×40 objective lens) using a mercury vapor lamp as the light source. We have used three different filters in the examination: FITC filter for green fluorescence color (with excitation at bandpass [BP] 475 ± 40 nm; emission BP 530 ± 50 nm), DsRed filter for orange fluorescence color (with excitation at BP 550 ± 25 nm; emission BP 605 ± 70 nm), and Alexa Fluor 660 filter for red fluorescence color (with excitation at BP 600 ± 50 nm; emission BP 685 ± 50 nm) [21].

### 2.4 MP analysis by FTIR

After fluorescence microscope examination, selected particles and fibers were further analyzed by FTIR for plastic particles’ differentiation. Spectra of FTIR were recorded in the range of 4,000–450 cm^−1^. OriginLab 2021 software was used to plot FTIR data. Finally, FTIR spectra were compared to the reference spectra of siMPle 2020 software (found in its database) to identify the type of the extracted MPs.

## 3 Results

### 3.1 MP identification using Nile red dye

**Figure 3:**
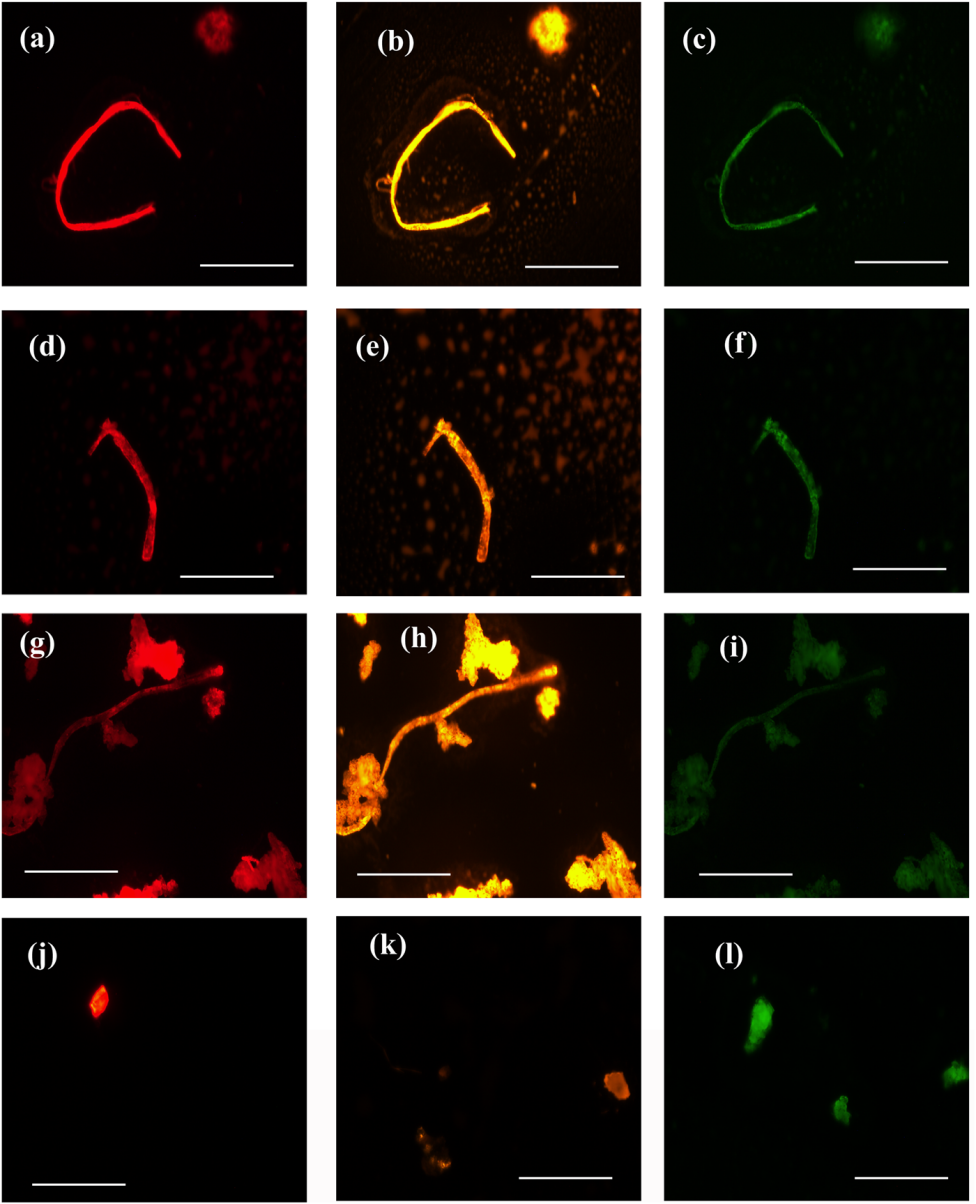
Fluorescence photomicrographs of MP fibers and small particles with three sets of excitation wave lengths after staining with Nile red in acetone at scale bar 100 µm.

In the present study, we have reported MP particles and fibers in muscles and gills’ samples from all the selected fish species (*n* = 22). Figure 3 shows fluorescence micrographs that are representative of MPs found in samples. In which, MPs with various types show different responses when using a set of fluorescence filters. The Nile red-stained MP particles/fibers were well recognized by red and orange fluorescence filters than the green ones. FTIR technique was further done to characterize MP type.

### 3.2 MP analysis by FTIR

**Figure 4:**
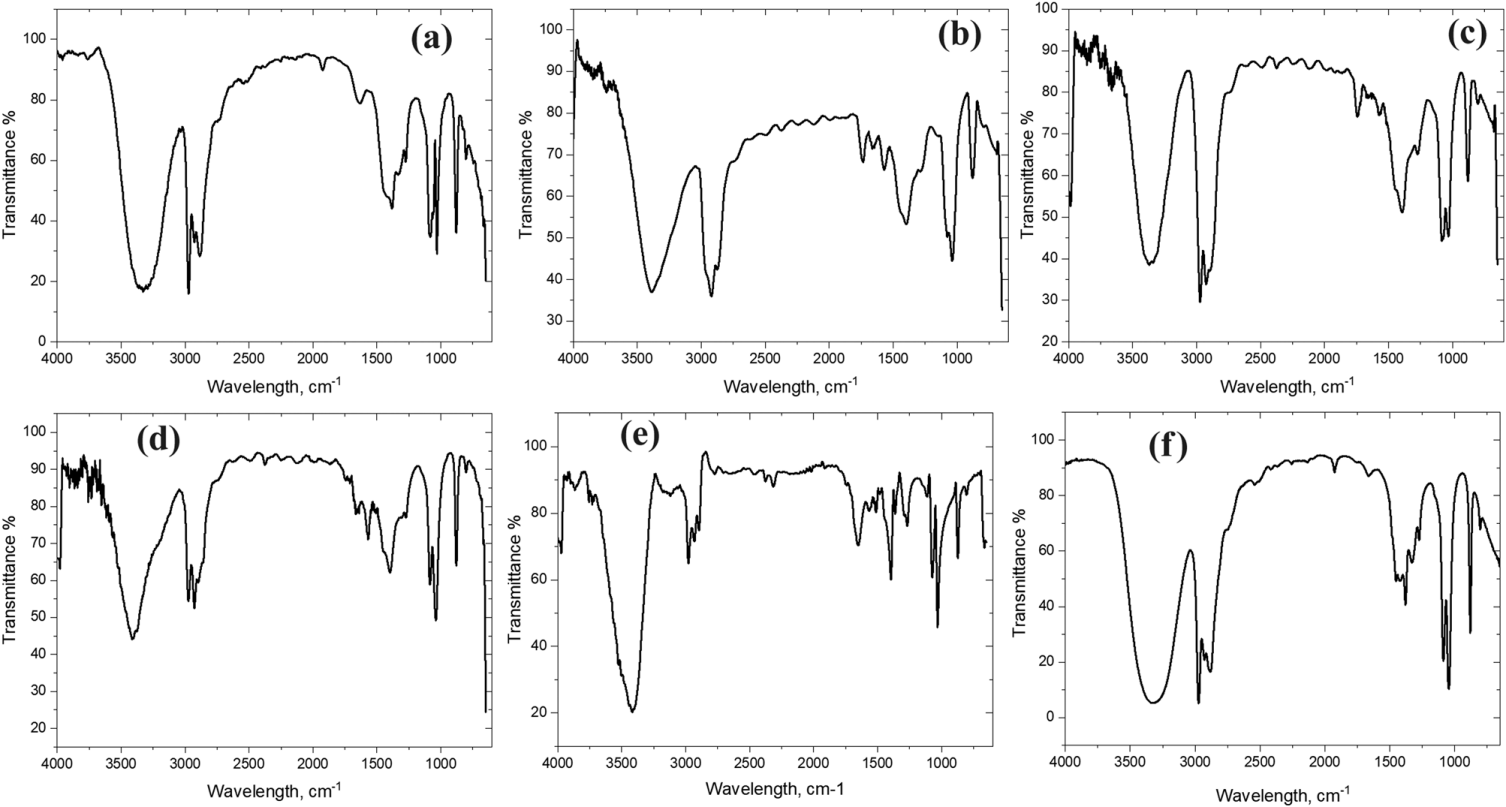
FTIR spectra of poly(2,4,6,-tribromostyrene) (a), CSPE (b and d), PVDF (c), PVB (e), and PVF (f) that are present in samples.

FTIR analyzes particles and fibers that were identified by Nile red dye to determine MP type. The FTIR spectra of most selected particles/fibers were identified as polymers after aligning with siMPle software reference data. We have found that MPs extracted from *Sardina* (epipelagic species) were poly(vinyl butyral) (PVB) and those extracted from *Mugil*, *Sphyraena*, *Thunnus*, and *Scomber* (pelagic species) were poly(vinylidene fluoride) (PVDF) and poly(2,4,6,-tribromostyrene) (PtBS). In addition, FTIR has identified MP particles and fibers present in demersal/benthopelagic species (*Trachurus*, *Chanos*, *Squalus*, *Pampus*, *Centropristis*, *Pagrus*, and *Netuma*) of the PtBS and chlorosulfonated polyethylene (CSPE) types. Moreover, *Mullus* and *Caranx* (benthic fish) samples contain PtBS particles. Reef-associated species, *Hemiramphus*, *Acanthopagrus*, *Saurida*, *Calotomus*, *Epinephelus*, and *Lethrinus*, have three different MP particles/fibers PtBS, PVDF, and poly(vinyl formal) (PVF). Finally, coastal inshore water (*Pomadasys*) and shallow water (*Oreochromis*) species’ samples contain PtBS. FTIR analysis of selected samples is shown in Figure 4.

### 3.3 MP characteristics

**Figure 5:**
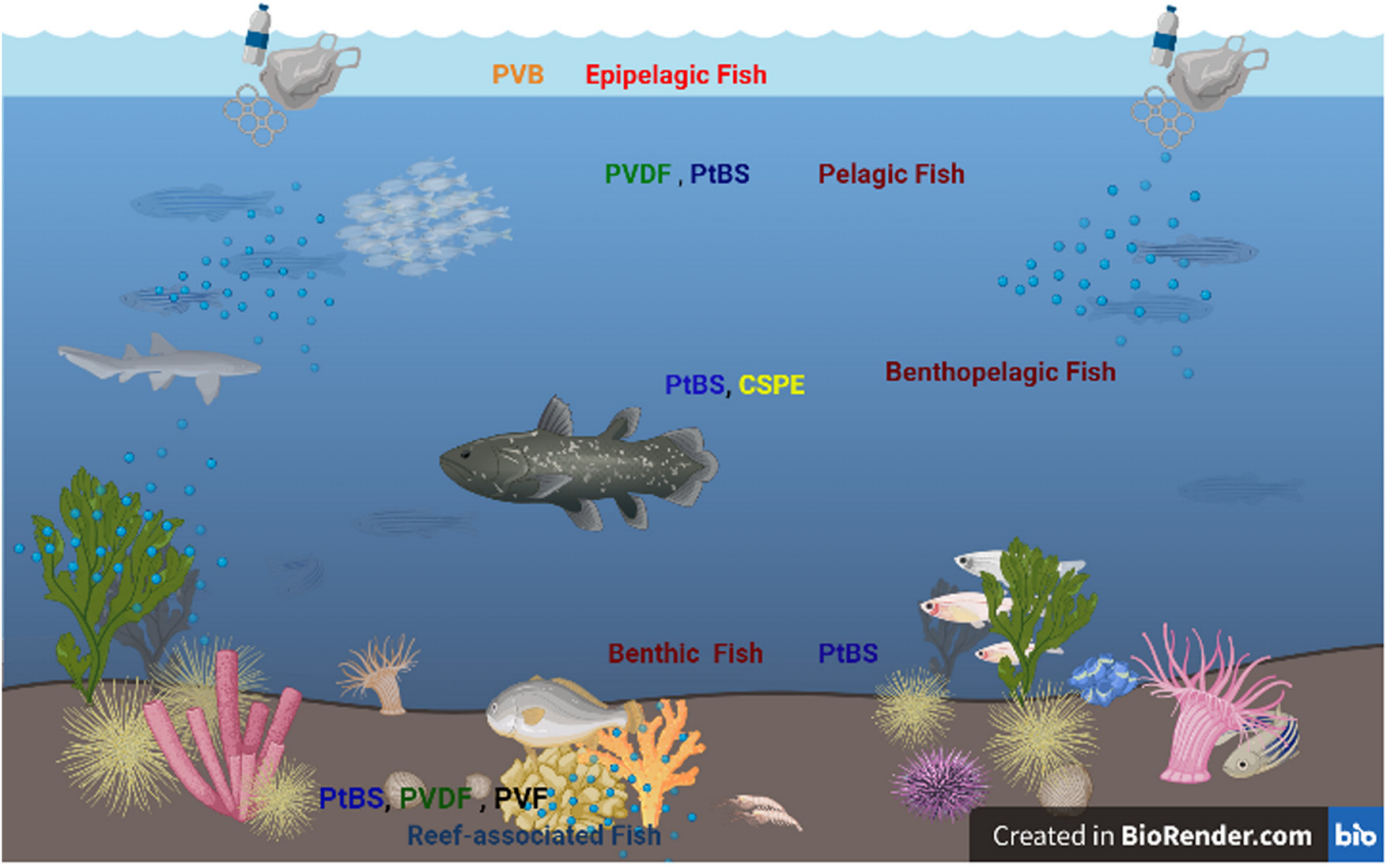
Schematic figure showing the distribution of MPs found in fish samples of the present study according to their marine habitat. Created with BioRender.com (2021).

The type of the extracted MPs and their size range per species are summarized in Table 2, in which the number of MPs in pooled samples of gills and muscles/species ranges from 1 to 10 particles/fiber. White color particles are the most abundant color of extracted MPs. In addition, the dominant type of MPs was particles rather than fibers or thread shape. The size of MPs ranges from 10 to 470 µm. In addition, it was recorded that the reef-associated species have higher MPs than species that are found near the surface such as shallow, coastal inshore waters, and epipelagic regions. Moreover, we have detected that the reef-associated species have more different types of MPs (PtBS, PVDF, and PVF) than others. On the other hand, there is a diversity in the number of extracted MPs found within pelagic, benthopelagic, and benthic species. Figure 5 shows a schematic photo for the distribution of MP particles and fibers present in the different 22 fish species according to their marine habitat.

**Table 2:** The type and number of MPs found in samples of the present study

Species name	Types of extracted MPs	Number of MPs in pooled samples of gills and muscles/species	Size range (µm)
*Hemiramphus far*	PtBS, PVDF, and PVF	6 particles	10–50
*Trachurus indicus*	PtBS and CSPE	1 fiber and 2 particles	10–20
*Pomadasys argenteus*	PtBS	2 particles	150–200
*Saurida undosquamis*	PtBS, PVDF, and PVF	6 particles	10–50
*Calotomus viridescens*	PtBS, PVDF, and PVF	1 fiber and 8 particles	20–150
*Acanthopagrus catenula*	PtBS, PVDF, and PVF	1 fiber and 3 particles	10–470
*Chanos chanos*	PtBS and CSPE	5 particles	30–80
*Sardina pilchardus*	PVB	1 fiber	170
*Mugil cephalus*	PVDF and PtBS	2 fibers and 4 particles	15–50
*Oreochromis spilurus*	PtBS	2 particles	150–200
*Mullus barbatus*	PtBS	3 particles	15–80
*Squalus acanthias*	PtBS and CSPE	6 particles	15–30
*Pampus argenteus*	PtBS and CSPE	7 particles	50–130
*Epinephelus morio*	PtBS, PVDF, and PVF	5 particles	20–120
*Sphyraena barracuda*	PVDF and PtBS	7 particles	30–100
*Lethrinus nebulosus*	PtBS, PVDF, and PVF	6 particles	10–50
*Centropristis striata*	PtBS and CSPE	5 particles	15–30
*Pagrus major*	PtBS and CSPE	6 particles	10–30
*Caranx caninus*	PtBS	2 particles	100–200
*Thunnus orientalis*	PVDF and PtBS	10 particles	10–70
*Scomber scombrus*	PVDF and PtBS	9 particles	10–60
*Netuma thalassina*	PtBS and CSPE	6 particles	10–30

The most abundant extracted MPs are white in color that their size ranges from 10 to 470 µm.

## 4 Discussion

Nowadays, there is a significant concern about MP contamination in both the aquatic environment and terrestrial biota, because MP was reported as being a vector for several pathogens, contaminants, and potentially toxic elements [22,23,24,25]. Therefore, several studies have focused on MP characterization and identification in the terrestrial and aquatic ecosystems to determine the severity of MP contamination [8,15,21]. However, the risk on human health due to MP bioaccumulation and biomagnification along the food web remains the primary aim for these numerous investigations [26].

We have chosen to evaluate MP pollution in gills (as an entrance for MPs through the water to other tissues) and muscle samples (the main part for human consumption) for 22 different fish species (*n* = 1). Most studies assessed MPs in the gastrointestinal tract of fish rather than other organs. However, in large fish, the internal viscera with the alimentary canal, gills, fins, head, and scales are removed before human consumption, but small pelagic fish are eaten as a whole [27,28]. We have detected MPs with different types in gills and muscle tissues of all the studied species (100% proportion). Our results, related to gills, were consistent with other recent studies that have assessed MP contamination in gills of different freshwater and marine fish species [29,30]. Park et al. [29] have reported MPs in gills and intestine of six freshwater fish species (*n* = 1 from each species) carp, crucian carp, bluegill, bass, catfish, and snakehead with a proportion (100%) higher than previous studies (ranges from 5 to 85%) [31,32,33]. They have concluded that high probability (100%) might return to the small sampling size (*n* = 1 per species) of their study.

On the other hand, there is slowly emerging evidence of MP occurrence in tissues other than the digestive tract in fish, such as the liver and muscles. We have detected MPs in different species of the present study in the muscle tissue by using Nile red dye. Collard et al. [34] have reported MPs contamination in the liver of the European anchovies (*Engraulis encrasicolus*); however, MPs were absent in the livers or muscles of other several important commercial species [29,30]. Akhbarizadeh et al. [35] have detected MPs in fish muscle without performing any polymer identification to determine their type.

According to the marine habitat of the selected studied species, we have found different MP particles/fibers in their gills and muscles. According to FTIR analysis followed by reference alignment, it was recorded that epipelagic sample contains PVB; pelagic species have PVDF and PtBS; demersal/benthopelagic species have PtBS and CSPE polymer types; benthic fish samples have PtBS; reef-associated species have PtBS, PVDF, and PVF; and coastal inshore water and shallow water species’ samples contain PtBS. We have categorized them according to habitat, not feeding habitat, because most of them have diverse meal types from planktons to small fish, worms, other marine invertebrates, and crustaceans. We have determined that PtBS, a type of polystyrene (PS) MPs, is the most widely distributed polymer type in the present study.

These findings are consistent with an earlier study by Baalkhuyur et al. [18]; they have reported MPs in the form of polypropylene, polyethylene, PS, polyvinyl chloride, and polyacrylonitrile by using FTIR spectroscopy in the alimentary canal of 18 different fish species along the Saudi Arabian Red Sea coast. On the contrary, the size of MPs found in samples of the present study is smaller in size (10–470 µm) than that they have reported (1–3 mm), because their samples are from the gut that does not reach muscle tissue yet. In addition, they have indicated that the mean MPs/fish from demersal, seagrass, and coral reef habitats were significantly higher than that in the mesopelagic habitat. In agreement with the present study, they have found a higher prevalence of MP debris in coral reef species (46.2%) than those in other habitats. They have suggested that species associated with reef habitats are more likely to ingest MPs than those found in other habitats. Moreover, the present study has recorded that the demersal species also have a high number of MPs with two different types (PtBS and CSPE), which was due to the variation in their feeding habitat of both plant and animal origins (such as crustaceans, benthic fish, mollusks, and algae). Baalkhuyur et al. [18] did not find a significant difference in the prevalence of MP ingestion across different habitats’ species that could be returned to their sampling size limitation. Jabeen et al. [36] have suggested that habitat, feeding habits, and feeding strategies could influence the likelihood of plastic debris ingestion regardless of the prey type.

The polymers that we have reported in the present study were found everywhere in our life. PVF and PVB polymers act as toughening agents to the brittle phenolic resins applied as coatings for metals [37,38]. CSPE, also known as HYPALON, is a synthetic rubber used in sports equipment, inflatable boats, roof coatings, and folding kayaks [39]. PVDF homopolymer is one of the toughest resins used in architectural coatings [40]. PS is a significant polymer litter on land and marine systems, mainly along shores and waterways, that are easily spread by wind [41,42,43]. PS is widely used due to its lightweight, high insulation value, and moisture resistance. It is employed for packaging, razors, containers, disposable dishes, and bottles. That is why PtBS is the most recorded polymer in most of the present studied species due to the wide use of PS [44]. Our study was done on fish species from the market; therefore, there is a need to investigate fresh samples from the marine source site, sediment, and water samples.

The main problem of MP accumulation in fish is their efficiency in adsorbing persistent organic pollutants found in the environment. In turn, MPs will be transferred through trophic levels and accumulated by a food chain until reaching the human body [45]. There is no apparent impact of MP ingestion on human health via seafood consumption, but they may pose a health risk to humans through physical and chemical pathways that should not be ignored in future research [46].

A limitation of this study is the low number of fish per species in our investigation, because we have decided to screen different species rather than increasing fish number per species. As we have referred, this is a preliminary screening that needs to be done on more samples.

## 5 Conclusion

Today, MPs became a global ecological problem either for the terrestrial environment or for the marine environment that led to increasing scientific concern on them. In this study, we focused on the selection of different fish species related to various marine habitats: epipelagic, pelagic, benthopelagic, benthic, reef-associated, and shallow water habitats for MP detection. Nile red dye is a simple way to detect MPs but not specific; therefore, FTIR analysis was used as a specific technique for MP type identification. Different previous research articles have detected MPs in gills and the alimentary canal of fish, but there is scarcity for this point related to the edible part such as muscles. MPs were determined and characterized in the gill and muscle samples from all the studied fish species in the present study. Different MPs were present, but PtBS as a type of PS was the most dominant one in most species with different habitats. Research studies with a larger sampling size from the source site will be critical for the ecotoxicology assessment of MP pollution of various organs of a range of fish species.
